# A Broad Examination of Mental Health Literacy Among College Students in the United States

**DOI:** 10.3928/24748307-20231023-01

**Published:** 2023-10

**Authors:** Susan M. Hannan, Tony T. Wells

## Abstract

**Background:**

High levels of mental health literacy (MHL) have been linked to the ability to correctly recognize certain problems as mental health issues, which then may lead to effective help-seeking behaviors. Most research on MHL has focused on a limited number of psychiatric diagnoses, using Australian samples.

**Objective:**

This study aimed to investigate various components of MHL in a large sample of undergraduate students in the United States.

**Methods:**

We conducted a vignette-based study with 843 undergraduate students. Six psychiatric diagnoses (and two “non-disordered” scenarios) were represented in distinct vignettes. Participants rated the severity of each vignette character's problem, the helpfulness of numerous treatment options, and the likelihood that different etiological factors contributed to the character's problem.

**Key Results:**

Across all clinical vignettes, therapy/counseling was perceived to be the most helpful treatment. Participants rated “personal weakness/lack of willpower” as contributing the most to the alcohol use disorder (AUD) character's problems. Our hypothesis related to how perceptions of etiology may impact participants' perceptions of different types of treatment was partially supported for the depression character. When participants described the depression character as having a “psychological/mental health problem,” they were more likely to perceive therapy/counseling as being helpful compared to medication.

**Conclusions:**

Participants recognized most of the psychiatric diagnoses as a mental health problem, acknowledged the seriousness of the presenting problems, and recommended effective help-seeking behavior. However, undergraduate U.S. students could benefit from increased MHL specifically related to AUD. [***HLRP: Health Literacy Research and Practice*. 2023;7(4):e207–e214.**]

## Abstract (plain language summary)

Plain Language Summary:

This study sought to measure various components of MHL in a large sample of U.S. undergraduate students. Findings demonstrated that AUD remained largely misunderstood and stigmatized. Students would likely benefit from increased education related to factors that contribute to alcohol use disorder.

Mental health literacy (MHL) has been defined as “knowledge and beliefs about mental disorders which aid their recognition, management, or prevention” (Jorm et al., 1997, p. 182). MHL is derived from the term “health literacy,” which describes an individual's ability to obtain, understand, and communicate health-related information to make informed decisions related to one's health (Berkman et al., 2010). MHL is comprised of several components, including but not limited to the ability to recognize specific psychiatric diagnoses, as well as knowledge and beliefs about the etiology of psychiatric symptoms and forms of interventions (Jorm, 2000). Previous research has suggested that early recognition of symptoms associated with psychiatric diagnoses may facilitate faster help-seeking behavior (such as psychotherapy), which in turn may lead to better mental health outcomes (Colizzi et al., 2020). Therefore, it is important to examine MHL in varying populations to determine potential gaps in knowledge that may hinder individuals' abilities to make informed decisions about their mental health.

One population that may be particularly vulnerable to mental health issues is college students. Due to various stressors associated with attending college (e.g., academic and financial pressures, gaining independence from family, forming new interpersonal relationships), it is not uncommon for students to experience the initial onset of mental health issues during the college years (Pedrelli et al., 2015). According to the American College Health Association (ACHA), within the last 12 months (of taking the survey) over 66% of undergraduate students felt overwhelming anxiety, over 46% felt so depressed it was difficult to function, and over 14% seriously considered suicide (ACHA, 2019). Furthermore, a recent meta-analysis suggested that U.S. college students (relative to college students in other countries) reported elevated symptoms of anxiety, depression, and sleep disturbances (Deng et al., 2021). Understanding MHL in this vulnerable population is therefore crucial.

Past research has shown that undergraduate students have difficulty recognizing various symptoms as mental health problem. For example, in a sample of 248 U.S. undergraduate students, Coles and Coleman (2010) found (using vignette methodology) that students were especially likely to label panic disorder as a “medical problem” and generalized anxiety disorder (GAD) as “general life stress” (Coles & Coleman, 2010). However, many students did provide accurate diagnostic labels to vignettes that depicted social phobia, obsessive-compulsive disorder (OCD), and depression (Coles & Coleman, 2010). Yap et al. (2013) also used vignette methodology to measure facets of MHL in Australian youth age 15 to 25 years). They found that only 10% of participants stated that “nothing would stop them” from seeking help for a hypothetical mental disorder and that feelings of embarrassment were one of the most commonly reported barriers to help-seeking (Yap et al., 2013).

Few studies have also examined whether different etiological attributions impact perceptions of treatment options. Iselin and Addis (2003) gave a sample of 36 undergraduate students and 36 mental health clients six vignettes depicting individuals with depression. The etiology of the presenting problem of the vignette characters was manipulated to suggest physical (i.e., biological) causal factors, psychological causal factors, or no etiological information was presented. Participants were instructed to rate the perceived helpfulness of different treatment options (e.g., psychological, medical) after reading each vignette. Iselin and Addis (2003) found that the perceived helpfulness of a treatment was related to causal beliefs about mental illness. In other words, participants rated psychotherapy as more helpful when they were presented with psychological causal information, whereas they rated biological treatments (e.g., medication) as more helpful when presented with physical causal factors. Furthermore, the undergraduate student participants rated treatments as significantly less helpful overall as compared to the mental health client participants. Results from the Iselin and Addis (2003) study suggest that perceptions of etiology impact how people perceive different types of treatment, which therefore may influence help-seeing behavior.

## The Current Study

The primary aim of the current study was to investigate various components of MHL (as conceptualized by Jorm's [1997] theoretical framework) in a large sample of undergraduate students from the U.S. Vignettes are a common tool for assessing MHL; in fact, the Mental Health Literacy Questionnaire (MHLQ) (Jorm et al., 1997) has been the most widely used vignette-based measurement tool of MHL. Therefore, the current study chose to measure MHL using a revised version of the MHLQ adapted from previous studies (e.g., Coles & Coleman, 2010; Jorm et al., 1997; Yap et al., 2013). A substantial portion of research on MHL has also been conducted with Australian samples (e.g., Jorm 1997; Pilkington et al., 2013; Thompson et al., 2008; Yap et al., 2013); therefore, those findings may or may not generalize to other populations. Interestingly, the U.S. is one of only two countries that allows pharmaceutical companies to advertise prescription medications directly to consumers. Previous research has shown that this direct-to-consumer advertising model can influence perceptions of medications, as well as help-seeking behavior (Huh & Becker, 2005). Therefore, individuals from countries that allow direct-to-consumer advertising may have different perceptions of the helpfulness of biological treatments for mental illnesses as compared to individuals from countries that do not allow direct-to-consumer advertising. Furthermore, previous research has focused predominately on understanding MHL as it relates to disorders of anxiety and depression. The current study therefore sought to assess MHL as it relates to several categories of psychiatric diagnoses, including anxiety, mood, psychotic, and substance use disorders.

The current study first sought to assess how undergraduate student participants judged the severity of each vignette character's problem. Additionally, we assessed whether participants believed that the vignette characters should get help for their problems. Participants also rated numerous treatment options (e.g., medication, therapy/counseling) on how helpful (or harmful) they thought the treatment might be for the problems described in the vignettes. Similarly, participants also rated numerous etiological factors (e.g., stress, genetics) on how likely it was that the factor contributed to the problem described in the vignette. Participants were then assessed on their ability to accurately describe each vignette character as having a psychological/mental health problem. Based on the framework that beliefs about etiology of a mental health disorder will impact how people perceive different types of treatment (including the helpfulness of different treatments; Iselin & Addis, 2003) findings from Iselin and Addis (2003), we predicted that endorsement of a vignette character as having a “medical/physical” problem would be associated with greater perceived helpfulness of biological treatment (i.e., medication) compared to psychological treatment (i.e., psychotherapy). Similarly, we also predicted that endorsement of a vignette character as having a “psychological/mental health” problem would be associated with greater perceived helpfulness of psychological treatment than biological treatment.

## Methods

### Participants

The current study recruited 1,041 undergraduate students from an Introduction to Psychology mass testing pool at a large southwestern university. Data from 198 participants needed to be removed after it was determined that those participants failed to accurately respond to numerous validity/attention check items, resulting in a total sample size of 843 participants. All participants were at least age 18 years (*M* = 19.23, standard deviation [*SD*] = 1.57, range = 18–29); 73.9% of the sample identified as women. Most participants self-identified as White (78.6%), whereas 6.3% self-identified as Native American, 5% as Black/African American, 4% as Hispanic, 2.8% as Asian American, and 2.7% as race and ethnicity not available. Regarding class year, 56.2% identified as freshmen, 23.8% as sophomores, 12.1% as juniors, 7.1% as seniors, and 0.7% as other. Most participants (84.2%) reported that they had never been diagnosed with or treated for any psychological or mental health issue.

### Vignettes

Participants were provided with several clinical vignettes presenting symptomology consistent with the following psychiatric diagnoses: social anxiety disorder (SAD), major depressive disorder (MDD), OCD, schizophrenia, alcohol use disorder (AUD), and GAD. The vignettes were not labeled with the diagnosis. We also included two “non-disordered” vignettes that depicted characters with general life problems (e.g., a character that is questioning her religion/stopped believing in God and a character that no longer sees his friends due to interest in new hobbies) that did not warrant a psychiatric diagnosis. All names included in each vignette are fictitious and do not reflect real patients. Participants were provided with the following instructions prior to reading and rating the vignettes: “Now, you will read 8 short vignettes of people experiencing certain problems or issues. Then you will answer questions about how you perceive the person's problems/issues. There is no one ‘right’ answer for each vignette, so please answer the questions based on what you think. Don't spend too long thinking about any one question. If you aren't sure about your answer, just go with your initial choice and move on.” See **Table A** for a full copy of the vignettes used in the current study.

**Table A.**
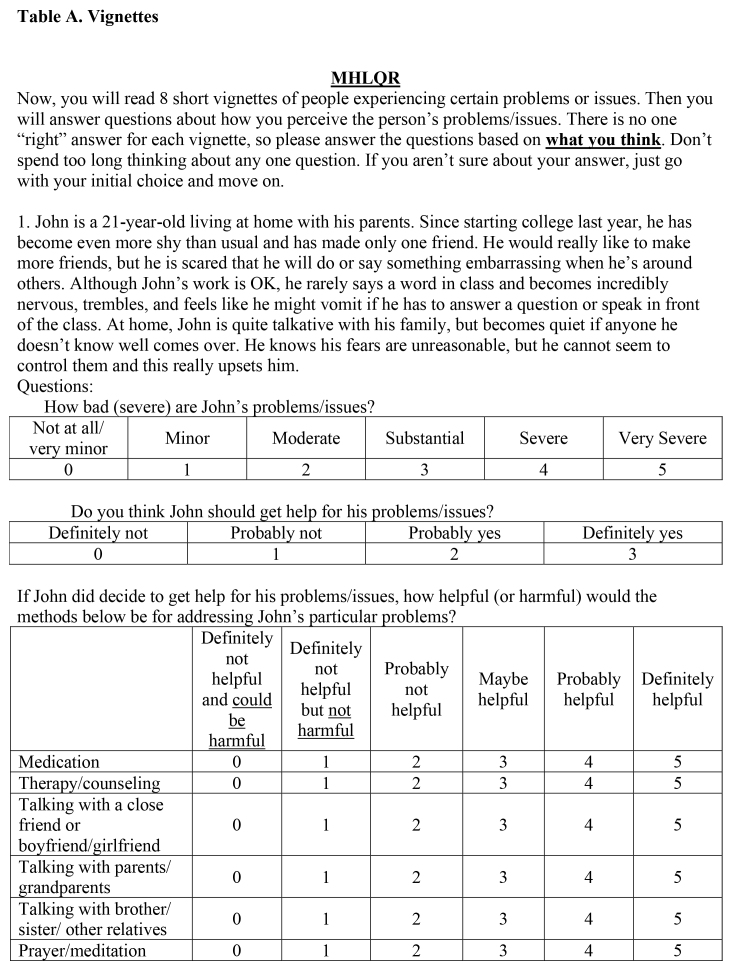
Vignettes

**MHLQR**
Now, you will read 8 short vignettes of people experiencing certain problems or issues. Then you will answer questions about how you perceive the person's problems/issues. There is no one “right” answer for each vignette, so please answer the questions based on **what you think**. Don't spend too long thinking about any one question. If you aren't sure about your answer, just go with your initial choice and move on.
1. John is a 21-year-old living at home with his parents. Since starting college last year, he has become even more shy than usual and has made only one friend. He would really like to make more friends, but he is scared that he will do or say something embarrassing when he's around others. Although John's work is OK, he rarely says a word in class and becomes incredibly nervous, trembles, and feels like he might vomit if he has to answer a question or speak in front of the class. At home, John is quite talkative with his family, but becomes quiet if anyone he doesn't know well comes over. He knows his fears are unreasonable, but he cannot seem to control them and this really upsets him.
Questions:
How bad (severe) are John's problems/issues? **Table:** Not at all/very minor Minor Moderate Substantial Severe Very Severe 0 1 2 3 4 5
Do you think John should get help for his problems/issues? **Table:** Definitely not Probably not Probably yes Definitely yes 0 1 2 3
If John did decide to get help for his problems/issues, how helpful (or harmful) would the methods below be for addressing John's particular problems? **Table:** Definitely not helpful and could be harmful Definitely not helpful but not harmful Probably not helpful Maybe helpful Probably helpful Definitely helpful Medication 0 1 2 3 4 5 Therapy/counseling 0 1 2 3 4 5 Talking with a close friend or boyfriend/girlfriend 0 1 2 3 4 5 Talking with parents/grandparents 0 1 2 3 4 5 Talking with brother/sister/other relatives 0 1 2 3 4 5 Prayer/meditation 0 1 2 3 4 5

**Table:**
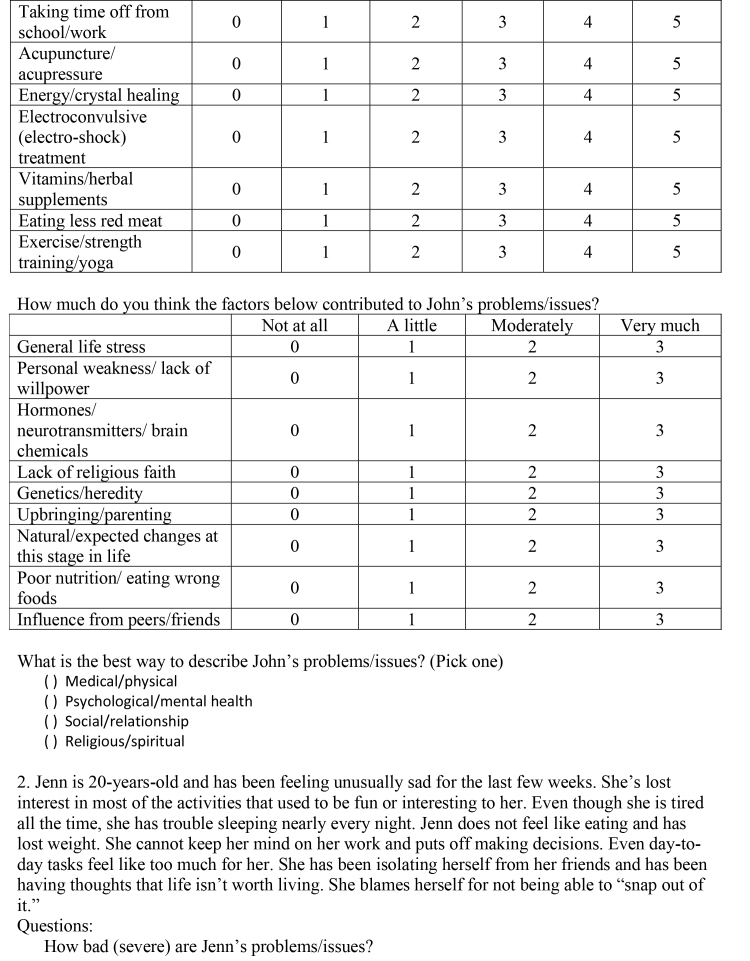


**Table:** Taking time off from school/work 0 1 2 3 4 5 Acupuncture/acupressure 0 1 2 3 4 5 Energy/crystal healing 0 1 2 3 4 5 Electroconvulsive (electro-shock) treatment 0 1 2 3 4 5 Vitamins/herbal supplements 0 1 2 3 4 5 Eating less red meat 0 1 2 3 4 5 Exercise/strength training/yoga 0 1 2 3 4 5
How much do you think the factors below contributed to John's problems/issues? **Table:** Not at all A little Moderately Very much General life stress 0 1 2 3 Personal weakness/lack of willpower 0 1 2 3 Hormones/neurotransmitters/brain chemicals 0 1 2 3 Lack of religious faith 0 1 2 3 Genetics/heredity 0 1 2 3 Upbringing/parenting 0 1 2 3 Natural/expected changes at this stage in life 0 1 2 3 Poor nutrition/eating wrong foods 0 1 2 3 Influence from peers/friends 0 1 2 3
What is the best way to describe John's problems/issues? (Pick one) ( ) Medical/physical ( ) Psychological/mental health ( ) Social/relationship ( ) Religious/spiritual
2. Jenn is 20-years-old and has been feeling unusually sad for the last few weeks. She's lost interest in most of the activities that used to be fun or interesting to her. Even though she is tired all the time, she has trouble sleeping nearly every night. Jenn does not feel like eating and has lost weight. She cannot keep her mind on her work and puts off making decisions. Even day-today tasks feel like too much for her. She has been isolating herself from her friends and has been having thoughts that life isn't worth living. She blames herself for not being able to “snap out of it.”
Questions:
How bad (severe) are Jenn's problems/issues?

**Table:**
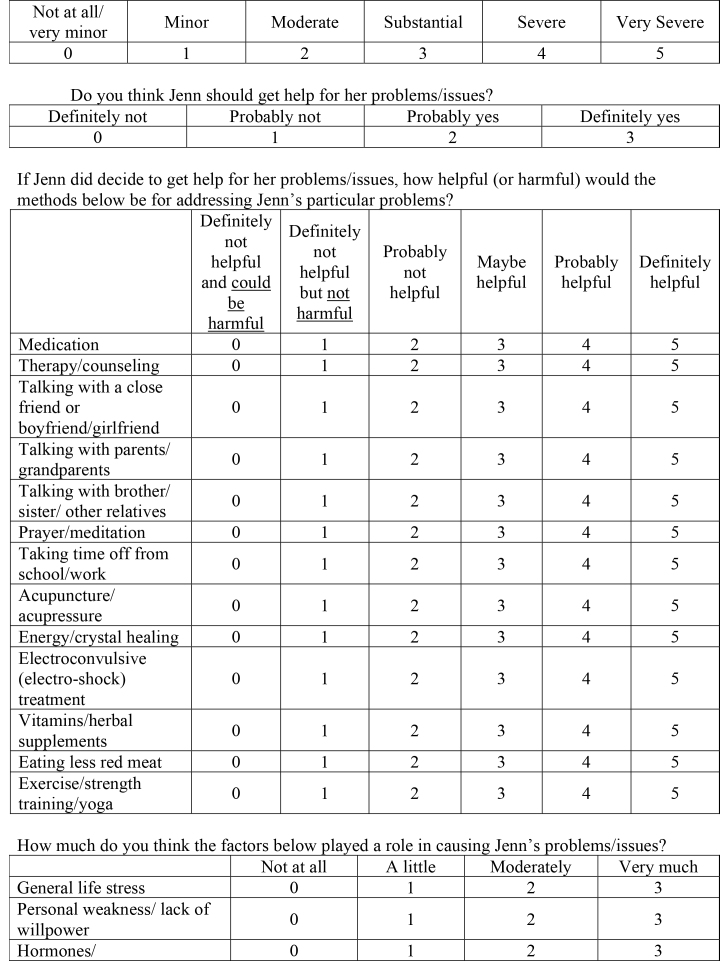


**Table:** Not at all/very minor Minor Moderate Substantial Severe Very Severe 0 1 2 3 4 5
Do you think Jenn should get help for her problems/issues? **Table:** Definitely not Probably not Probably yes Definitely yes 0 1 2 3
If Jenn did decide to get help for her problems/issues, how helpful (or harmful) would the methods below be for addressing Jenn's particular problems? **Table:** Definitely not helpful and could be harmful Definitely not helpful but not harmful Probably not helpful Maybe helpful Probably helpful Definitely helpful Medication 0 1 2 3 4 5 Therapy/counseling 0 1 2 3 4 5 Talking with a close friend or boyfriend/girlfriend 0 1 2 3 4 5 Talking with parents/grandparents 0 1 2 3 4 5 Talking with brother/sister/other relatives 0 1 2 3 4 5 Prayer/meditation 0 1 2 3 4 5 Taking time off from school/work 0 1 2 3 4 5 Acupuncture/acupressure 0 1 2 3 4 5 Energy/crystal healing 0 1 2 3 4 5 Electroconvulsive (electro-shock) treatment 0 1 2 3 4 5 Vitamins/herbal supplements 0 1 2 3 4 5 Eating less red meat 0 1 2 3 4 5 Exercise/strength training/yoga 0 1 2 3 4 5
How much do you think the factors below played a role in causing Jenn's problems/issues? **Table:** Not at all A little Moderately Very much General life stress 0 1 2 3 Personal weakness/lack of willpower 0 1 2 3 Hormones/ 0 1 2 3

**Table:**
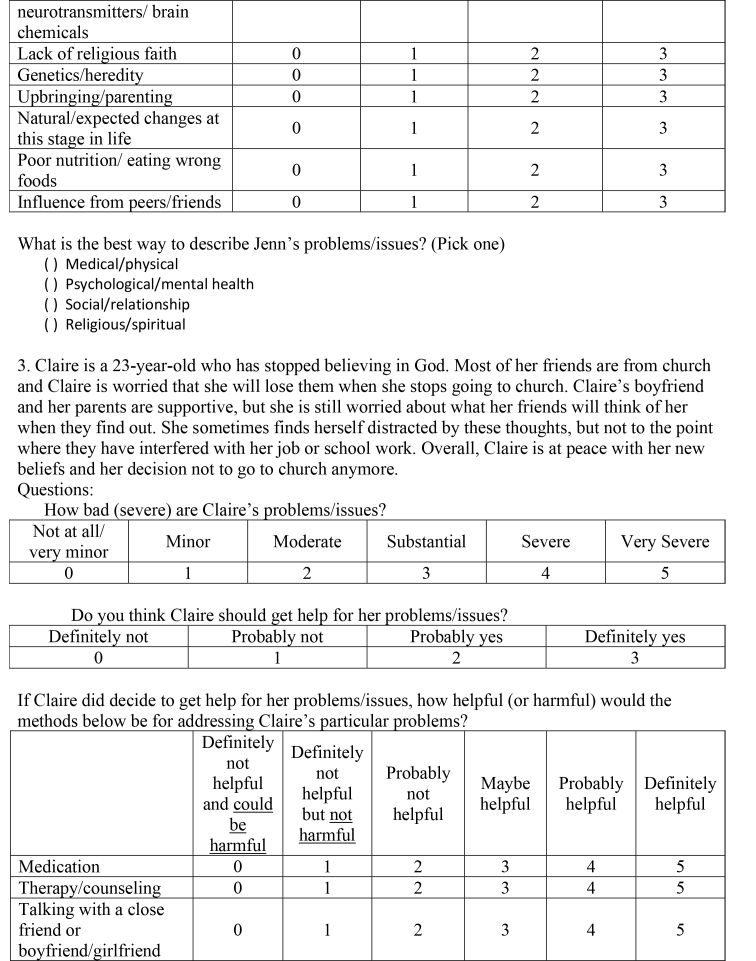


**Table:** neurotransmitters/brain chemicals Lack of religious faith 0 1 2 3 Genetics/heredity 0 1 2 3 Upbringing/parenting 0 1 2 3 Natural/expected changes at this stage in life 0 1 2 3 Poor nutrition/eating wrong foods 0 1 2 3 Influence from peers/friends 0 1 2 3
What is the best way to describe Jenn's problems/issues? (Pick one) ( ) Medical/physical ( ) Psychological/mental health ( ) Social/relationship ( ) Religious/spiritual
3. Claire is a 23-year-old who has stopped believing in God. Most of her friends are from church and Claire is worried that she will lose them when she stops going to church. Claire's boyfriend and her parents are supportive, but she is still worried about what her friends will think of her when they find out. She sometimes finds herself distracted by these thoughts, but not to the point where they have interfered with her job or school work. Overall, Claire is at peace with her new beliefs and her decision not to go to church anymore.
Questions:
How bad (severe) are Claire's problems/issues? **Table:** Not at all/very minor Minor Moderate Substantial Severe Very Severe 0 1 2 3 4 5
Do you think Claire should get help for her problems/issues? **Table:** Definitely not Probably not Probably yes Definitely yes 0 1 2 3
If Claire did decide to get help for her problems/issues, how helpful (or harmful) would the methods below be for addressing Claire's particular problems? **Table:** Definitely not helpful and could be harmful Definitely not but not helpful harmful Probably not helpful Maybe helpful Probably helpful Definitely helpful Medication 0 1 2 3 4 5 Therapy/counseling 0 1 2 3 4 5 Talking with a close friend or boyfriend/girlfriend 0 1 2 3 4 5

**Table:**
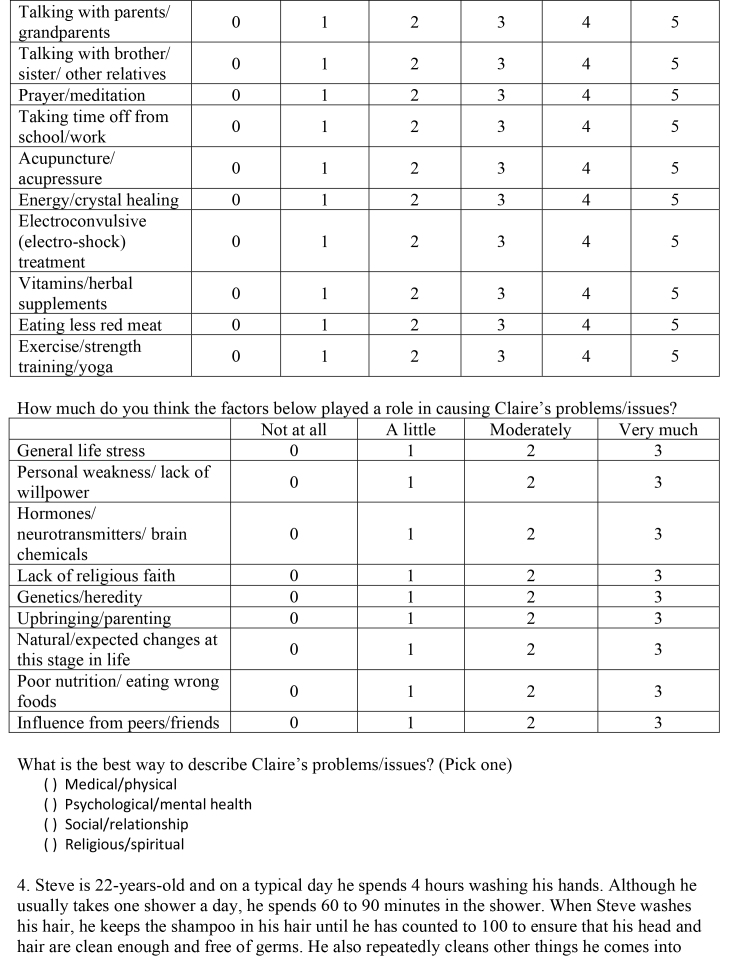


**Table:** Talking with parents/grandparents 0 1 2 3 4 5 Talking with brother/sister/other relatives 0 1 2 3 4 5 Prayer/meditation 0 1 2 3 4 5 Taking time off from school/work 0 1 2 3 4 5 Acupuncture/acupressure 0 1 2 3 4 5 Energy/crystal healing 0 1 2 3 4 5 Electroconvulsive (electro-shock) treatment 0 1 2 3 4 5 Vitamins/herbal supplements 0 1 2 3 4 5 Eating less red meat 0 1 2 3 4 5 Exercise/strength training/yoga 0 1 2 3 4 5
How much do you think the factors below played a role in causing Claire's problems/issues? **Table:** Not at all A little Moderately Very much General life stress 0 1 2 3 Personal weakness/lack of willpower 0 1 2 3 Hormones/neurotransmitters/brain chemicals 0 1 2 3 Lack of religious faith 0 1 2 3 Genetics/heredity 0 1 2 3 Upbringing/parenting 0 1 2 3 Natural/expected changes at this stage in life 0 1 2 3 Poor nutrition/eating wrong foods 0 1 2 3 Influence from peers/friends 0 1 2 3
What is the best way to describe Claire's problems/issues? (Pick one) ( ) Medical/physical ( ) Psychological/mental health ( ) Social/relationship ( ) Religious/spiritual
4. Steve is 22-years-old and on a typical day he spends 4 hours washing his hands. Although he usually takes one shower a day, he spends 60 to 90 minutes in the shower. When Steve washes his hair, he keeps the shampoo in his hair until he has counted to 100 to ensure that his head and hair are clean enough and free of germs. He also repeatedly cleans other things he comes into

**Table:**
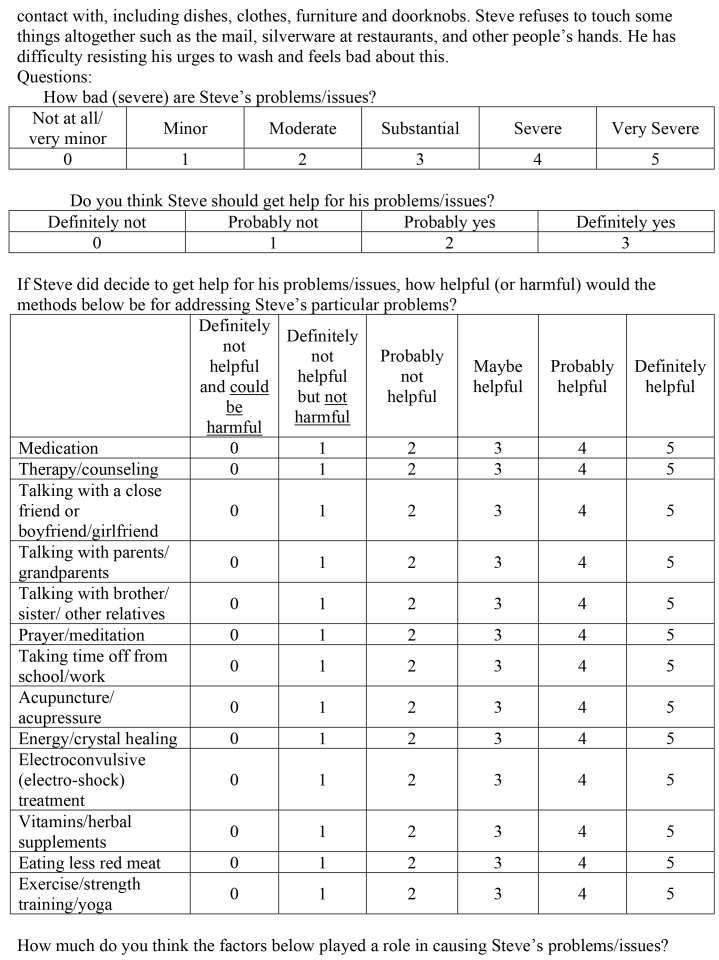


contact with, including dishes, clothes, furniture and doorknobs. Steve refuses to touch some things altogether such as the mail, silverware at restaurants, and other people's hands. He has difficulty resisting his urges to wash and feels bad about this.
Questions:
How bad (severe) are Steve's problems/issues? **Table:** Not at all/very minor Minor Moderate Substantial Severe Very Severe 0 1 2 3 4 5
Do you think Steve should get help for his problems/issues? **Table:** Definitely not Probably not Probably yes Definitely yes 0 1 2 3
If Steve did decide to get help for his problems/issues, how helpful (or harmful) would the methods below be for addressing Steve's particular problems? **Table:** Definitely not helpful and could be harmful Definitely not helpful but not harmful Probably not helpful Maybe helpful Probably helpful Definitely helpful Medication 0 1 2 3 4 5 Therapy/counseling 0 1 2 3 4 5 Talking with a close friend or boyfriend/girlfriend 0 1 2 3 4 5 Talking with parents/grandparents 0 1 2 3 4 5 Talking with brother/sister/other relatives 0 1 2 3 4 5 Prayer/meditation 0 1 2 3 4 5 Taking time off from school/work 0 1 2 3 4 5 Acupuncture/acupressure 0 1 2 3 4 5 Energy/crystal healing 0 1 2 3 4 5 Electroconvulsive (electro-shock) treatment 0 1 2 3 4 5 Vitamins/herbal supplements 0 1 2 3 4 5 Eating less red meat 0 1 2 3 4 5 Exercise/strength training/yoga 0 1 2 3 4 5
How much do you think the factors below played a role in causing Steve's problems/issues?

**Table:**
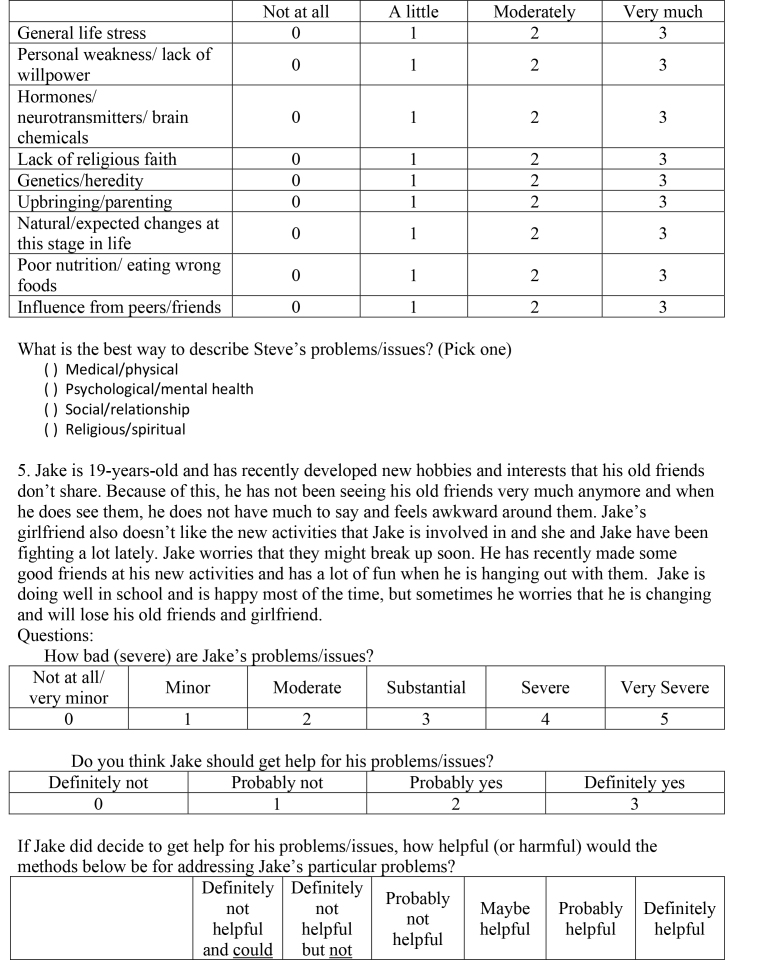


**Table:** Not at all A little Moderately Very much General life stress 0 1 2 3 Personal weakness/lack of willpower 0 1 2 3 Hormones/neurotransmitters/brain chemicals 0 1 2 3 Lack of religious faith 0 1 2 3 Genetics/heredity 0 1 2 3 Upbringing/parenting 0 1 2 3 Natural/expected changes at this stage in life 0 1 2 3 Poor nutrition/eating wrong foods 0 1 2 3 Influence from peers/friends 0 1 2 3
What is the best way to describe Steve's problems/issues? (Pick one) ( ) Medical/physical ( ) Psychological/mental health ( ) Social/relationship ( ) Religious/spiritual
5. Jake is 19-years-old and has recently developed new hobbies and interests that his old friends don't share. Because of this, he has not been seeing his old friends very much anymore and when he does see them, he does not have much to say and feels awkward around them. Jake's girlfriend also doesn't like the new activities that Jake is involved in and she and Jake have been fighting a lot lately. Jake worries that they might break up soon. He has recently made some good friends at his new activities and has a lot of fun when he is hanging out with them. Jake is doing well in school and is happy most of the time, but sometimes he worries that he is changing and will lose his old friends and girlfriend.
Questions:
How bad (severe) are Jake's problems/issues? **Table:** Not at all/very minor Minor Moderate Substantial Severe Very Severe 0 1 2 3 4 5
Do you think Jake should get help for his problems/issues? **Table:** Definitely not Probably not Probably yes Definitely yes 0 1 2 3
If Jake did decide to get help for his problems/issues, how helpful (or harmful) would the methods below be for addressing Jake's particular problems? **Table:** Definitely not helpful and could Definitely not helpful but not Probably not helpful Maybe helpful Probably helpful Definitely helpful

**Table:**
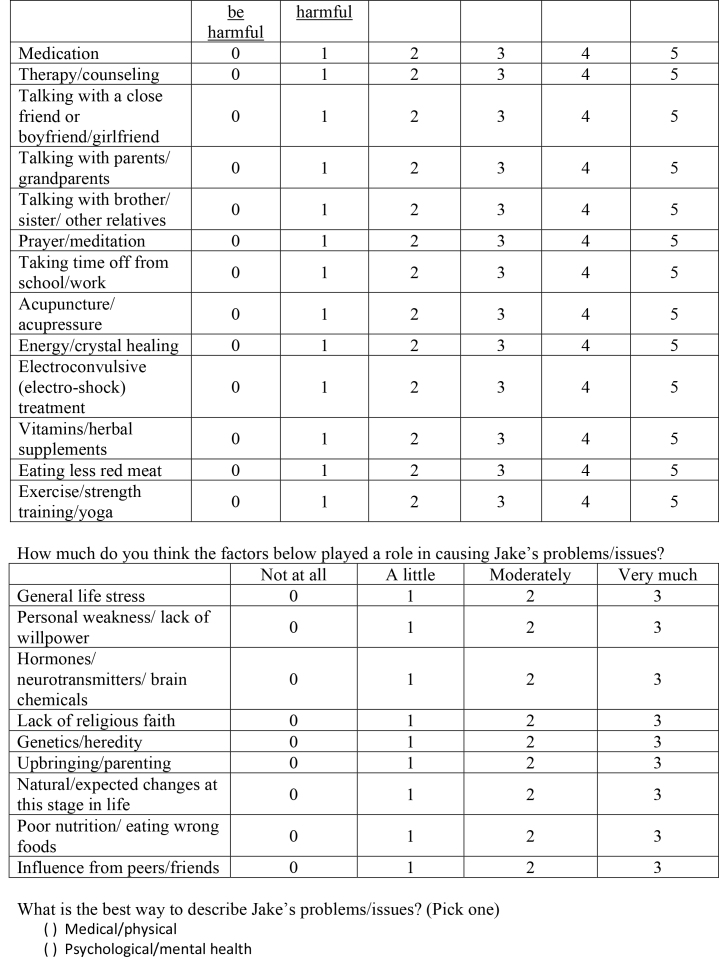


**Table:** be harmful harmful Medication 0 1 2 3 4 5 Therapy/counseling 0 1 2 3 4 5 Talking with a close friend or boyfriend/girlfriend 0 1 2 3 4 5 Talking with parents/grandparents 0 1 2 3 4 5 Talking with brother/sister/other relatives 0 1 2 3 4 5 Prayer/meditation 0 1 2 3 4 5 Taking time off from school/work 0 1 2 3 4 5 Acupuncture/acupressure 0 1 2 3 4 5 Energy/crystal healing 0 1 2 3 4 5 Electroconvulsive (electro-shock) treatment 0 1 2 3 4 5 Vitamins/herbal supplements 0 1 2 3 4 5 Eating less red meat 0 1 2 3 4 5 Exercise/strength training/yoga 0 1 2 3 4 5
How much do you think the factors below played a role in causing Jake's problems/issues? **Table:** Not at all A little Moderately Very much General life stress 0 1 2 3 Personal weakness/lack of willpower 0 1 2 3 Hormones/neurotransmitters/brain chemicals 0 1 2 3 Lack of religious faith 0 1 2 3 Genetics/heredity 0 1 2 3 Upbringing/parenting 0 1 2 3 Natural/expected changes at this stage in life 0 1 2 3 Poor nutrition/eating wrong foods 0 1 2 3 Influence from peers/friends 0 1 2 3
What is the best way to describe Jake's problems/issues? (Pick one) ( ) Medical/physical ( ) Psychological/mental health

**Table:**
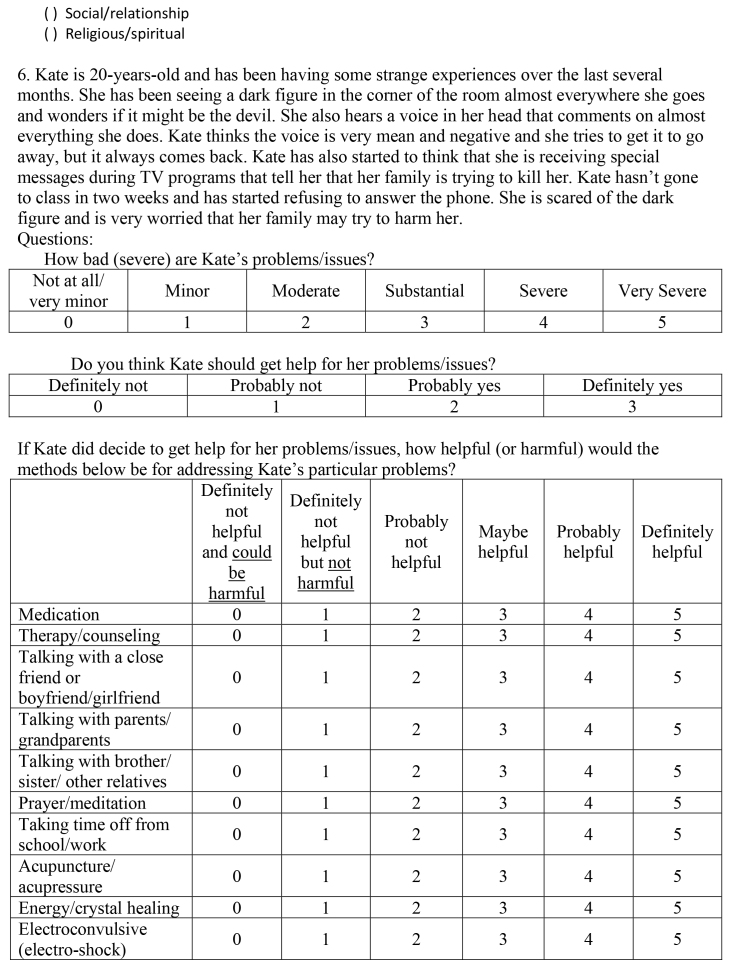


( ) Social/relationship ( ) Religious/spiritual
6. Kate is 20-years-old and has been having some strange experiences over the last several months. She has been seeing a dark figure in the corner of the room almost everywhere she goes and wonders if it might be the devil. She also hears a voice in her head that comments on almost everything she does. Kate thinks the voice is very mean and negative and she tries to get it to go away, but it always comes back. Kate has also started to think that she is receiving special messages during TV programs that tell her that her family is trying to kill her. Kate hasn't gone to class in two weeks and has started refusing to answer the phone. She is scared of the dark figure and is very worried that her family may try to harm her.
Questions:
How bad (severe) are Kate's problems/issues? **Table:** Not at all/very minor Minor Moderate Substantial Severe Very Severe 0 1 2 3 4 5
Do you think Kate should get help for her problems/issues? **Table:** Definitely not Probably not Probably yes Definitely yes 0 1 2 3
If Kate did decide to get help for her problems/issues, how helpful (or harmful) would the methods below be for addressing Kate's particular problems? **Table:** Definitely not helpful and could be harmful Definitely not helpful but not harmful Probably not helpful Maybe helpful Probably helpful Definitely helpful Medication 0 1 2 3 4 5 Therapy/counseling 0 1 2 3 4 5 Talking with a close friend or boyfriend/girlfriend 0 1 2 3 4 5 Talking with parents/grandparents 0 1 2 3 4 5 Talking with brother/sister/other relatives 0 1 2 3 4 5 Prayer/meditation 0 1 2 3 4 5 Taking time off from school/work 0 1 2 3 4 5 Acupuncture/acupressure 0 1 2 3 4 5 Energy/crystal healing 0 1 2 3 4 5 Electroconvulsive (electro-shock) 0 1 2 3 4 5

**Table:**
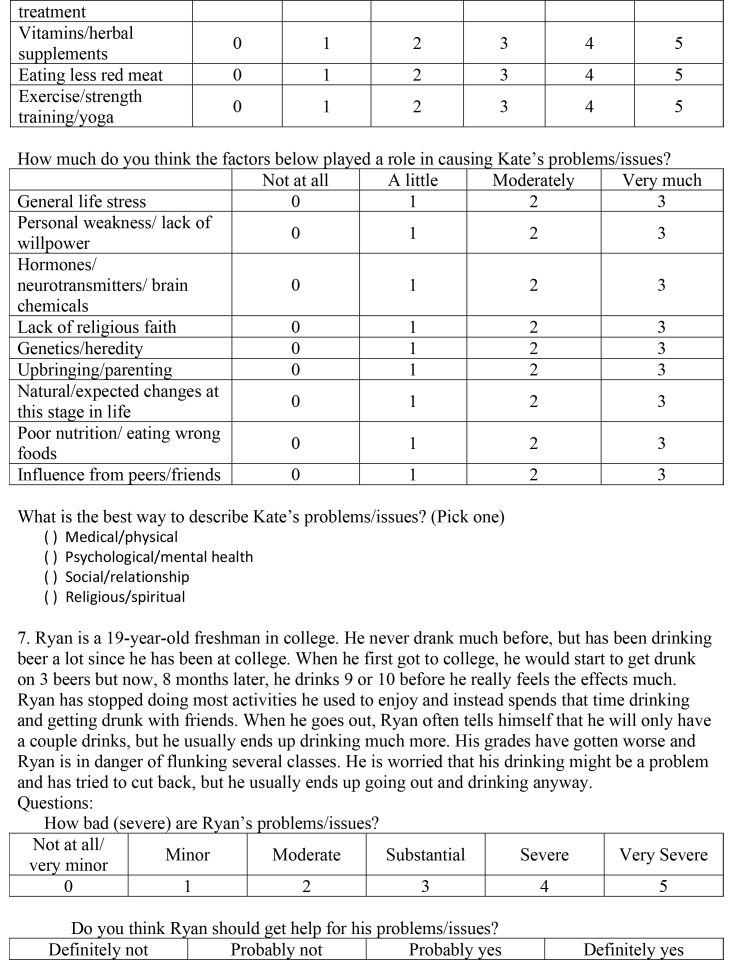


**Table:** treatment Vitamins/herbal supplements 0 1 2 3 4 5 Eating less red meat 0 1 2 3 4 5 Exercise/strength training/yoga 0 1 2 3 4 5
How much do you think the factors below played a role in causing Kate's problems/issues? **Table:** Not at all A little Moderately Very much General life stress 0 1 2 3 Personal weakness/lack of willpower 0 1 2 3 Hormones/neurotransmitters/brain chemicals 0 1 2 3 Lack of religious faith 0 1 2 3 Genetics/heredity 0 1 2 3 Upbringing/parenting 0 1 2 3 Natural/expected changes at this stage in life 0 1 2 3 Poor nutrition/eating wrong foods 0 1 2 3 Influence from peers/friends 0 1 2 3
What is the best way to describe Kate's problems/issues? (Pick one) ( ) Medical/physical ( ) Psychological/mental health ( ) Social/relationship ( ) Religious/spiritual
7. Ryan is a 19-year-old freshman in college. He never drank much before, but has been drinking beer a lot since he has been at college. When he first got to college, he would start to get drunk on 3 beers but now, 8 months later, he drinks 9 or 10 before he really feels the effects much. Ryan has stopped doing most activities he used to enjoy and instead spends that time drinking and getting drunk with friends. When he goes out, Ryan often tells himself that he will only have a couple drinks, but he usually ends up drinking much more. His grades have gotten worse and Ryan is in danger of flunking several classes. He is worried that his drinking might be a problem and has tried to cut back, but he usually ends up going out and drinking anyway.
Questions:
How bad (severe) are Ryan's problems/issues? **Table:** Not at all/very minor Minor Moderate Substantial Severe Very Severe 0 1 2 3 4 5
Do you think Ryan should get help for his problems/issues? **Table:** Definitely not Probably not Probably yes Definitely yes

**Table:**
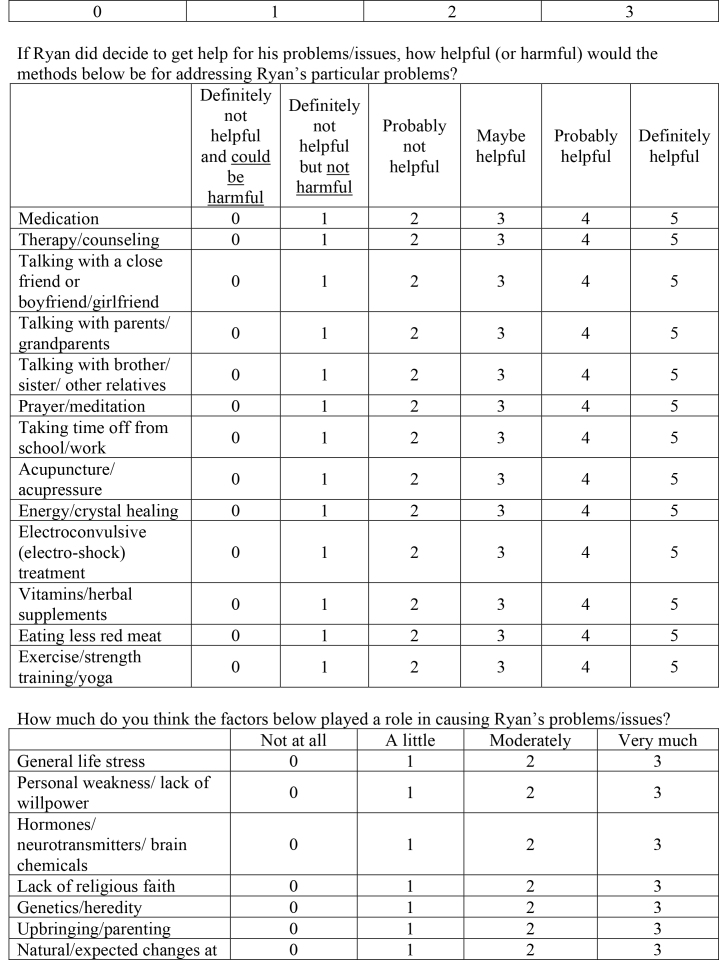


**Table:** 0 1 2 3
If Ryan did decide to get help for his problems/issues, how helpful (or harmful) would the methods below be for addressing Ryan's particular problems? **Table:** Definitely not helpful and could be harmful Definitely not helpful but not harmful Probably not helpful Maybe helpful Probably helpful Definitely helpful Medication 0 1 2 3 4 5 Therapy/counseling 0 1 2 3 4 5 Talking with a close friend or boyfriend/girlfriend 0 1 2 3 4 5 Talking with parents/grandparents 0 1 2 3 4 5 Talking with brother/sister/other relatives 0 1 2 3 4 5 Prayer/meditation 0 1 2 3 4 5 Taking time off from school/work 0 1 2 3 4 5 Acupuncture/acupressure 0 1 2 3 4 5 Energy/crystal healing 0 1 2 3 4 5 Electroconvulsive (electro-shock) treatment 0 1 2 3 4 5 Vitamins/herbal supplements 0 1 2 3 4 5 Eating less red meat 0 1 2 3 4 5 Exercise/strength training/yoga 0 1 2 3 4 5
How much do you think the factors below played a role in causing Ryan's problems/issues? **Table:** Not at all A little Moderately Very much General life stress 0 1 2 3 Personal weakness/lack of willpower 0 1 2 3 Hormones/neurotransmitters/brain chemicals 0 1 2 3 Lack of religious faith 0 1 2 3 Genetics/heredity 0 1 2 3 Upbringing/parenting 0 1 2 3 Natural/expected changes at 0 1 2 3

**Table:**
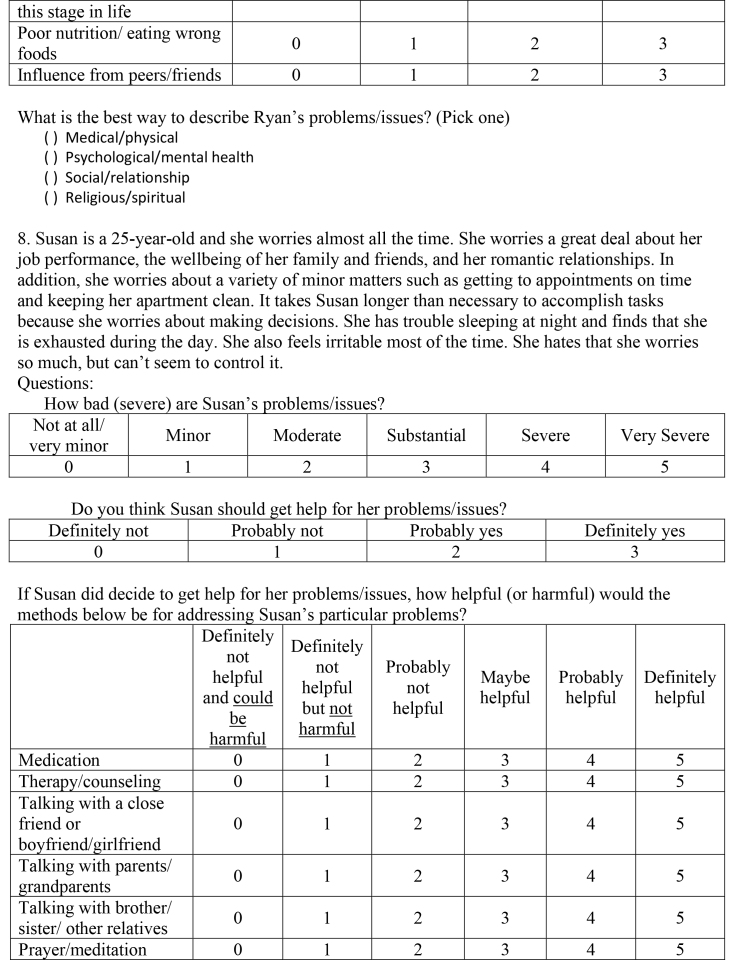


**Table:** this stage in life Poor nutrition/eating wrong foods 0 1 2 3 Influence from peers/friends 0 1 2 3
What is the best way to describe Ryan's problems/issues? (Pick one) ( ) Medical/physical ( ) Psychological/mental health ( ) Social/relationship ( ) Religious/spiritual
8. Susan is a 25-year-old and she worries almost all the time. She worries a great deal about her job performance, the wellbeing of her family and friends, and her romantic relationships. In addition, she worries about a variety of minor matters such as getting to appointments on time and keeping her apartment clean. It takes Susan longer than necessary to accomplish tasks because she worries about making decisions. She has trouble sleeping at night and finds that she is exhausted during the day. She also feels irritable most of the time. She hates that she worries so much, but can't seem to control it.
Questions:
How bad (severe) are Susan's problems/issues? **Table:** Not at all/very minor Minor Moderate Substantial Severe Very Severe 0 1 2 3 4 5
Do you think Susan should get help for her problems/issues? **Table:** Definitely not Probably not Probably yes Definitely yes 0 1 2 3
If Susan did decide to get help for her problems/issues, how helpful (or harmful) would the methods below be for addressing Susan's particular problems? **Table:** Definitely not helpful and could be harmful Definitely not helpful but not harmful Probably not helpful Maybe helpful Probably helpful Definitely helpful Medication 0 1 2 3 4 5 Therapy/counseling 0 1 2 3 4 5 Talking with a close friend or boyfriend/girlfriend 0 1 2 3 4 5 Talking with parents/grandparents 0 1 2 3 4 5 Talking with brother/sister/other relatives 0 1 2 3 4 5 Prayer/meditation 0 1 2 3 4 5

**Table:**
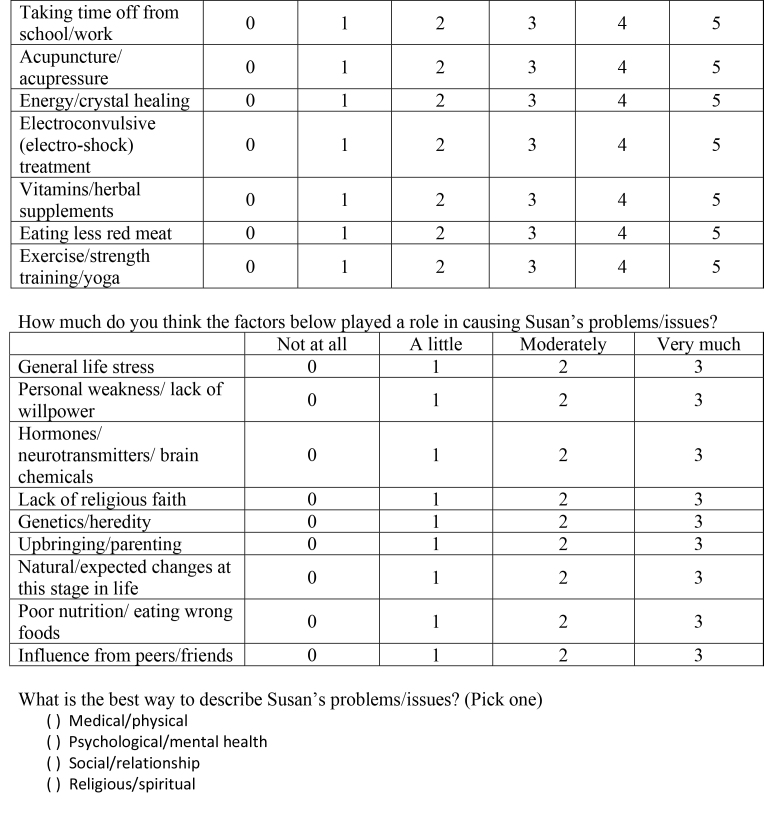


**Table:** Taking time off from school/work 0 1 2 3 4 5 Acupuncture/acupressure 0 1 2 3 4 5 Energy/crystal healing 0 1 2 3 4 5 Electroconvulsive (electro-shock) treatment 0 1 2 3 4 5 Vitamins/herbal supplements 0 1 2 3 4 5 Eating less red meat 0 1 2 3 4 5 Exercise/strength training/yoga 0 1 2 3 4 5
How much do you think the factors below played a role in causing Susan's problems/issues? **Table:** Not at all A little Moderately Very much General life stress 0 1 2 3 Personal weakness/lack of willpower 0 1 2 3 Hormones/neurotransmitters/brain chemicals 0 1 2 3 Lack of religious faith 0 1 2 3 Genetics/heredity 0 1 2 3 Upbringing/parenting 0 1 2 3 Natural/expected changes at this stage in life 0 1 2 3 Poor nutrition/eating wrong foods 0 1 2 3 Influence from peers/friends 0 1 2 3
What is the best way to describe Susan's problems/issues? (Pick one) ( ) Medical/physical ( ) Psychological/mental health ( ) Social/relationship ( ) Religious/spiritual

The vignettes, etiologies, and treatments and outcome measures used for this study were based on prior work using similarly structured vignettes (e.g., Coles & Coleman, 2010; Jorm et al., 1997; Yap et al., 2013). However, unlike previous research that used forced-choice response styles for etiology and treatment options within vignettes, the current study allowed participants to rate every etiological factor and treatment option that was presented to them. In other words, instead of forcing participants to categorically select one etiological and treatment option, we provided participants with a rating scale for every option (allowing for a more continuous assessment of these variables).

## Measures

### Demographics

Participants provided demographic information such as gender, age, year in college, race and ethnicity, and history of diagnosis/treatment of a mental health issue.

***Vignette problem severity.*** Perceived severity of the presenting problem was evaluated using a single item containing a 6-point scale from *not at all/very minor* to *very severe*.

***Vignette help-seeking endorsement. ***Attitudes about whether the person in the vignette should seek help for the problem described in the vignette were evaluated using a single item containing a 4-point scale from *definitely not* to *definitely yes*.

***Vignette treatment endorsement***. To evaluate perceptions of treatments, each treatment method was rated for perceived helpfulness/harmfulness on the following 6-point scale: *definitely not helpful and could be harmful*, *definitely not helpful but not harmful*, *probably not helpful*, *maybe helpful*, *probably helpful*, and *definitely helpful*. The treatment methods were presented in the following fixed order: medication, therapy/counseling, talking with a close friend or boyfriend/girlfriend, talking with parents/grandparents, talking with brother/sister/other relatives, prayer/meditation, taking time off from school/work, acupuncture/acupressure, energy/crystal healing, electroconvulsive (ECT) treatment, vitamins/herbal supplements, eating less red meat, and exercise/strength training/yoga.

***Vignette etiology endorsement.*** Perceptions about the causes for each vignette's presenting problem were evaluated by rating different etiological factors on a 4-point scale indicating the likelihood of a given etiological factor contributing to the problem (ranging from *not at all* to *very much*). The etiological factors were presented in the following fixed order: general life stress, personal weakness/lack of willpower, hormones/neurotransmitters/brain chemicals, lack of religious faith, genetics/hereditary, up-bringing/parenting, natural/expected changes at this stage in life, poor nutrition/eating wrong food, and influence from peers/friends. Additionally, participants were forced to select one of the following etiological descriptions to describe the presenting problem: medical/physical, psychological/mental health, social/relationship, or religious/spiritual.

### Procedure

Participants completed the study measures online as part of a mass testing procedure for course credit. Vignettes were presented in the following fixed order: SAD, MDD, first “non-disordered” vignette, OCD, second “non-disordered” vignette, schizophrenia, AUD, and GAD. Participants responded to each vignette by rating the severity of the problems, whether the participant would recommend the person in the vignette seek help, ratings of treatment, and ratings of etiologies. All participants provided informed consent prior to participation and all procedures were approved by the Oklahoma State University Institutional Review Board. All data were naturally observed (i.e., no manipulated conditions were present).

### Data Analysis

Data were analyzed using IBM SPSS Statistics 27.0. Data were first examined for out-of-range values, missing values, and normality. Frequency distributions were analyzed to assess participants' ratings of the severity of each vignette character's problems, treatment helpfulness, and the likelihood of etiological factors contributing to the problems. We also ran a frequency distribution to determine the way in which participants described each vignette character's problems/issues. Finally, we ran six 2 × 2 mixed factorial analysis of variances (ANOVAs) to assess whether participants' description of each vignette character's problem was related to perceived helpfulness of medication and therapy/counseling. Perceived treatment helpfulness was the dependent variable (rated on a 6-point scale from *definitely not helpful* and *could be harmful* to *definitely helpful*) with treatment type (medication, therapy/counseling) as the repeated measures factor and description of the problem (medical/physical vs. psychological/mental health) as the between subjects factor. Due to running six separate ANOVAs and observing unequal sample sizes in many of the cells, we chose a conservative alpha level of *p* < .001 to determine statistical significance. We analyzed the main effects and interaction effects; however, we did not interpret significant main effects if the interaction effect was also significant.

## Results

See **Table 1** for participant ratings to the question pertaining to the perceived severity of each vignette character's problems/issues. Notably, for the clinical vignettes, participants rated the schizophrenia character's problems as most severe and the SAD and GAD characters' problems as least severe. **Table 2** describes participant ratings to the question pertaining to whether each vignette character should get help for their problems. For the clinical vignettes, participants again rated the schizophrenia character as needing the most help and the SAD and GAD characters as needing the least help.

**Table 1:**
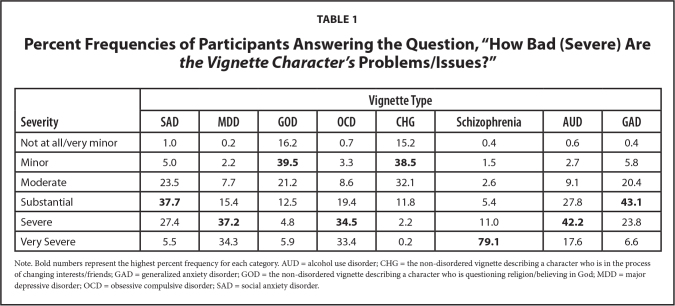
Percent Frequencies of Participants Answering the Question, “How Bad (Severe) Are *the Vignette Character's* Problems/Issues?”

**Severity**	**Vignette Type**							
	**SAD**	**MDD**	**GOD**	**OCD**	**CHG**	**Schizophrenia**	**AUD**	**GAD**
Not at all/very minor	1.0	0.2	16.2	0.7	15.2	0.4	0.6	0.4
Minor	5.0	2.2	**39.5**	3.3	**38.5**	1.5	2.7	5.8
Moderate	23.5	7.7	21.2	8.6	32.1	2.6	9.1	20.4
Substantial	**37.7**	15.4	12.5	19.4	11.8	5.4	27.8	**43.1**
Severe	27.4	**37.2**	4.8	**34.5**	2.2	11.0	**42.2**	23.8
Very Severe	5.5	34.3	5.9	33.4	0.2	**79.1**	17.6	6.6

Note. Bold numbers represent the highest percent frequency for each category. AUD = alcohol use disorder; CHG = the non-disordered vignette describing a character who is in the process of changing interests/friends; GAD = generalized anxiety disorder; GOD = the non-disordered vignette describing a character who is questioning religion/believing in God; MDD = major depressive disorder; OCD = obsessive compulsive disorder; SAD = social anxiety disorder.

**Table 2:**
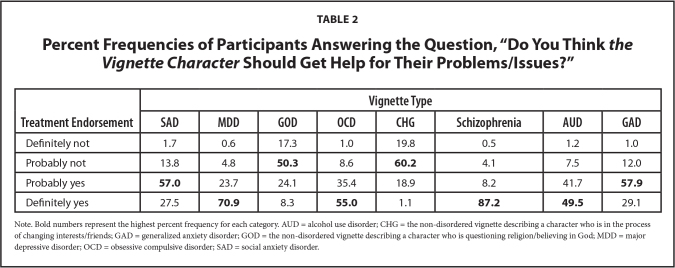
Percent Frequencies of Participants Answering the Question, “Do You Think *the Vignette Character* Should Get Help for Their Problems/Issues?”

**Treatment Endorsement**	**Vignette Type**							
	**SAD**	**MDD**	**GOD**	**OCD**	**CHG**	**Schizophrenia**	**AUD**	**GAD**
Definitely not	1.7	0.6	17.3	1.0	19.8	0.5	1.2	1.0
Probably not	13.8	4.8	**50.3**	8.6	**60.2**	4.1	7.5	12.0
Probably yes	**57.0**	23.7	24.1	35.4	18.9	8.2	41.7	**57.9**
Definitely yes	27.5	**70.9**	8.3	**55.0**	1.1	**87.2**	**49.5**	29.1

Note. Bold numbers represent the highest percent frequency for each category. AUD = alcohol use disorder; CHG = the non-disordered vignette describing a character who is in the process of changing interests/friends; GAD = generalized anxiety disorder; GOD = the non-disordered vignette describing a character who is questioning religion/believing in God; MDD = major depressive disorder; OCD = obsessive compulsive disorder; SAD = social anxiety disorder.

Perceived method of treatment helpfulness was rated on a scale from 0 to 5; we treated this variable as a continuous variable in all subsequent analyses. **Table 3** lists the means and standard deviations of perceived treatment helpfulness by vignette type. Across all of the clinical vignettes participants rated “therapy/counseling” as being the most helpful treatment method. For the non-disordered vignettes, “talking with a close friend/significant other” was rated as most helpful. Across all eight vignettes, ECT was rated as the least helpful treatment method.

**Table 3:**
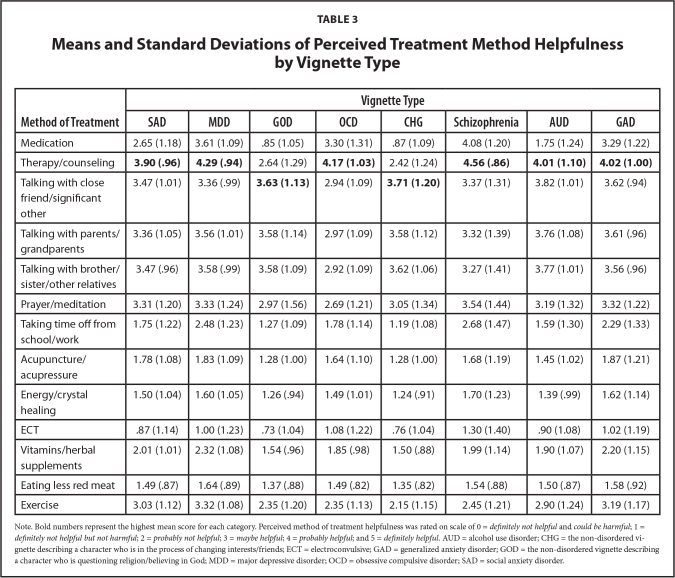
Means and Standard Deviations of Perceived Treatment Method Helpfulness by Vignette Type

**Method of Treatment**	**Vignette Type**							
	**SAD**	**MDD**	**GOD**	**OCD**	**CHG**	**Schizophrenia**	**AUD**	**GAD**
Medication	2.65 (1.18)	3.61 (1.09)	.85 (1.05)	3.30 (1.31)	.87 (1.09)	4.08 (1.20)	1.75 (1.24)	3.29 (1.22)
Therapy/counseling	**3.90 (.96)**	**4.29 (.94)**	2.64 (1.29)	**4.17 (1.03)**	2.42 (1.24)	**4.56 (.86)**	**4.01 (1.10)**	**4.02 (1.00)**
Talking with close friend/significant other	3.47 (1.01)	3.36 (.99)	**3.63 (1.13)**	2.94 (1.09)	**3.71 (1.20)**	3.37 (1.31)	3.82 (1.01)	3.62 (.94)
Talking with parents/grandparents	3.36 (1.05)	3.56 (1.01)	3.58 (1.14)	2.97 (1.09)	3.58 (1.12)	3.32 (1.39)	3.76 (1.08)	3.61 (.96)
Talking with brother/sister/other relatives	3.47 (.96)	3.58 (.99)	3.58 (1.09)	2.92 (1.09)	3.62 (1.06)	3.27 (1.41)	3.77 (1.01)	3.56 (.96)
Prayer/meditation	3.31 (1.20)	3.33 (1.24)	2.97 (1.56)	2.69 (1.21)	3.05 (1.34)	3.54 (1.44)	3.19 (1.32)	3.32 (1.22)
Taking time off from school/work	1.75 (1.22)	2.48 (1.23)	1.27 (1.09)	1.78 (1.14)	1.19 (1.08)	2.68 (1.47)	1.59 (1.30)	2.29 (1.33)
Acupuncture/acupressure	1.78 (1.08)	1.83 (1.09)	1.28 (1.00)	1.64 (1.10)	1.28 (1.00)	1.68 (1.19)	1.45 (1.02)	1.87 (1.21)
Energy/crystal healing	1.50 (1.04)	1.60 (1.05)	1.26 (.94)	1.49 (1.01)	1.24 (.91)	1.70 (1.23)	1.39 (.99)	1.62 (1.14)
ECT	.87 (1.14)	1.00 (1.23)	.73 (1.04)	1.08 (1.22)	.76 (1.04)	1.30 (1.40)	.90 (1.08)	1.02 (1.19)
Vitamins/herbal supplements	2.01 (1.01)	2.32 (1.08)	1.54 (.96)	1.85 (.98)	1.50 (.88)	1.99 (1.14)	1.90 (1.07)	2.20 (1.15)
Eating less red meat	1.49 (.87)	1.64 (.89)	1.37 (.88)	1.49 (.82)	1.35 (.82)	1.54 (.88)	1.50 (.87)	1.58 (.92)
Exercise	3.03 (1.12)	3.32 (1.08)	2.35 (1.20)	2.35 (1.13)	2.15 (1.15)	2.45 (1.21)	2.90 (1.24)	3.19 (1.17)

Note. Bold numbers represent the highest mean score for each category. Perceived method of treatment helpfulness was rated on scale of 0 = *definitely not helpful* and *could be harmful*; 1 = *definitely not helpful but not harmful*; 2 = *probably not helpful*; 3 = *maybe helpful*; 4 = *probably helpful*; and 5 = *definitely helpful*. AUD = alcohol use disorder; CHG = the non-disordered vignette describing a character who is in the process of changing interests/friends; ECT = electroconvulsive; GAD = generalized anxiety disorder; GOD = the non-disordered vignette describing a character who is questioning religion/believing in God; MDD = major depressive disorder; OCD = obsessive compulsive disorder; SAD = social anxiety disorder.

Perceived contributing factors related to each vignette character's problems was rated on a scale of 0 to 3; we treated this variable as a continuous variable in all subsequent analyses. **Table 4** lists the means and standard deviations of perceived contributing factors to each vignette characters' problems. Participants rated “life stress” as contributing the most to the SAD, MDD, and GAD characters' problems. “Hormones/neurotransmitters/brain chemicals” were rated as contributing the most to the OCD and Schizophrenia characters' problems. Participants rated “poor nutrition” as contributing the least to all vignette characters' problems except the MDD character. Participants rated “lack of religious faith” as contributing the least to the MDD character's problems. Finally, participants rated “personal weakness/lack of willpower” as contributing the most to the AUD character's problems.

**Table 4:**
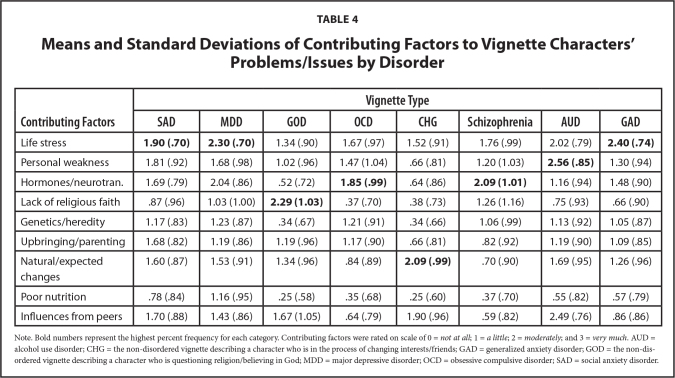
Means and Standard Deviations of Contributing Factors to Vignette Characters' Problems/Issues by Disorder

**Contributing Factors**	**Vignette Type**							
	**SAD**	**MDD**	**GOD**	**OCD**	**CHG**	**Schizophrenia**	**AUD**	**GAD**
Life stress	**1.90 (.70)**	**2.30 (.70)**	1.34 (.90)	1.67 (.97)	1.52 (.91)	1.76 (.99)	2.02 (.79)	**2.40 (.74)**
Personal weakness	1.81 (.92)	1.68 (.98)	1.02 (.96)	1.47 (1.04)	.66 (.81)	1.20 (1.03)	**2.56 (.85)**	1.30 (.94)
Hormones/neurotran.	1.69 (.79)	2.04 (.86)	.52 (.72)	**1.85 (.99)**	.64 (.86)	**2.09 (1.01)**	1.16 (.94)	1.48 (.90)
Lack of religious faith	.87 (.96)	1.03 (1.00)	**2.29 (1.03)**	.37 (.70)	.38 (.73)	1.26 (1.16)	.75 (.93)	.66 (.90)
Genetics/heredity	1.17 (.83)	1.23 (.87)	.34 (.67)	1.21 (.91)	.34 (.66)	1.06 (.99)	1.13 (.92)	1.05 (.87)
Upbringing/parenting	1.68 (.82)	1.19 (.86)	1.19 (.96)	1.17 (.90)	.66 (.81)	.82 (.92)	1.19 (.90)	1.09 (.85)
Natural/expected changes	1.60 (.87)	1.53 (.91)	1.34 (.96)	.84 (.89)	**2.09 (.99)**	.70 (.90)	1.69 (.95)	1.26 (.96)
Poor nutrition	.78 (.84)	1.16 (.95)	.25 (.58)	.35 (.68)	.25 (.60)	.37 (.70)	.55 (.82)	.57 (.79)
Influences from peers	1.70 (.88)	1.43 (.86)	1.67 (1.05)	.64 (.79)	1.90 (.96)	.59 (.82)	2.49 (.76)	.86 (.86)

Note. Bold numbers represent the highest percent frequency for each category. Contributing factors were rated on scale of 0 = *not at all*; 1 = *a little*; 2 = *moderately*; and 3 = *very much*. AUD = alcohol use disorder; CHG = the non-disordered vignette describing a character who is in the process of changing interests/friends; GAD = generalized anxiety disorder; GOD = the non-disordered vignette describing a character who is questioning religion/believing in God; MDD = major depressive disorder; OCD = obsessive compulsive disorder; SAD = social anxiety disorder.

When asked to describe the clinical vignette character's problems/issues, most participants did not label the SAD or AUD characters as having a mental health problem. In fact, only 17.6% of participants labeled the AUD character as having a “psychological/mental health” problem. Many participants (50.5%) reported that the AUD character's problem was “social/relationship” in nature. For the SAD vignette, 64.1% of participants labeled the character as having a “social/relationship” problem, whereas 31.1% of participants labeled the character as having a “psychological/mental health” problem. A large majority of participants (73.2%–80.2%) labeled the MDD, OCD, schizophrenia, and GAD characters as having a “psychological/mental health” problem.

Finally, we ran six 2 × 2 mixed factorials ANOVAs to assess whether participants' description of each clinical vignette character's problem was associated with perceived helpfulness of medication and therapy/counseling. Only significant results in line with our hypotheses are reported below (see **Table 5** for the complete ANOVA results). Our hypotheses were only (partially) supported for the MDD vignette. Based on the results of the 2 x 2 mixed ANOVA, there was a significant interaction effect (treatment type and description of the problem), *F*(1,729) = 15.63, *p* < .001, η*p*^2^ = .021. Partially in line with our prediction, a simple slopes analysis revealed that when participants described the MDD character as having a “psychological/mental health problem,” they were more likely to perceive therapy/counseling as being helpful (*M* = 4.49, *SD* = .71) compared to medication (*M* = 3.72, *SD* = 1.04).

**Table 5:**
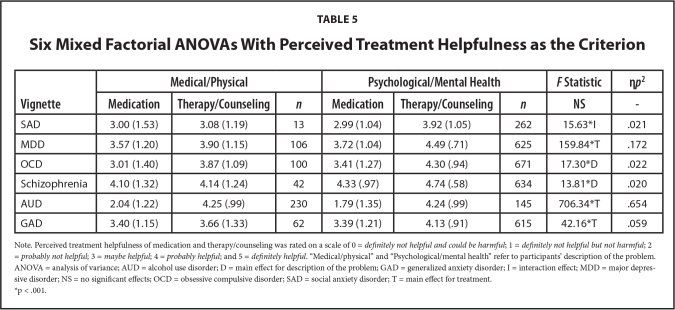
Six Mixed Factorial ANOVAs With Perceived Treatment Helpfulness as the Criterion

**Vignette**	**Medical/Physical**			**Psychological/Mental Health**			***F* Statistic**	**η*p*^2^**
	**Medication**	**Therapy/Counseling**	***n***	**Medication**	**Therapy/Counseling**	***n***	**NS**	**-**
SAD	3.00 (1.53)	3.08 (1.19)	13	2.99 (1.04)	3.92 (1.05)	262	15.63^*^I	.021
MDD	3.57 (1.20)	3.90 (1.15)	106	3.72 (1.04)	4.49 (.71)	625	159.84^*^T	.172
OCD	3.01 (1.40)	3.87 (1.09)	100	3.41 (1.27)	4.30 (.94)	671	17.30^*^D	.022
Schizophrenia	4.10 (1.32)	4.14 (1.24)	42	4.33 (.97)	4.74 (.58)	634	13.81^*^D	.020
AUD	2.04 (1.22)	4.25 (.99)	230	1.79 (1.35)	4.24 (.99)	145	706.34^*^T	.654
GAD	3.40 (1.15)	3.66 (1.33)	62	3.39 (1.21)	4.13 (.91)	615	42.16^*^T	.059

Note. Perceived treatment helpfulness of medication and therapy/counseling was rated on a scale of 0 = *definitely not helpful and could be harmful*; 1 = *definitely not helpful but not harmful*; 2 = *probably not helpful*; 3 = *maybe helpful*; 4 = *probably helpful*; and 5 = *definitely helpful*. “Medical/physical” and “Psychological/mental health” refer to participants' description of the problem. ANOVA = analysis of variance; AUD = alcohol use disorder; D = main effect for description of the problem; GAD = generalized anxiety disorder; I = interaction effect; MDD = major depressive disorder; NS = no significant effects; OCD = obsessive compulsive disorder; SAD = social anxiety disorder; T = main effect for treatment.

* p < .001.

## Discussion

The primary aim of the current study was to assess various components of MHL in a large undergraduate student sample. Most participants did not label neither the SAD nor the AUD vignette characters as having a “psychological/mental health” problem. Additionally, participants perceived the SAD and GAD vignette characters' problems to be less severe compared to all other vignettes. That said, participants in the current study did not attribute the SAD character's problems to “personal weakness/lack of willpower” to the extent found in previous research (e.g., Coles & Coleman, 2010). Unfortunately, participants in the current study rated “personal weakness/lack of willpower” as contributing the most to the AUD character's problems. While this finding likely highlights the continued stigma associated with AUD, it is interesting to note that compared to all other disorders, participants were most likely to describe the AUD character's problems as being “medical/physical” in nature. In other words, some participants reported perceiving the AUD character's problems to be due to a “personal weakness/lack of willpower,” while at the same time recognizing that the problems were “medical/physical” in nature. A recent review found that individuals were more likely to attribute the cause of AUD to that of personal weakness/responsibility as compared to MDD and Schizophrenia (Kilian et al., 2021). In fact, Kilian et al. (2021) suggest that AUD remains the most stigmatized mental health disorder.

Our predictions related to the relationship between participants' description of each vignette character's problem and perceived treatment helpfulness of medication and therapy/counseling were partially supported for the MDD vignette. Participants who described the MDD character as having a “psychological/mental health” problem were more likely to perceive therapy/counseling as being helpful compared to medication. However, participants who described the MDD character as having a “medical/physical” problem perceived medication and therapy/counseling to be equally helpful. No other interaction effects were significant for the remaining five clinical vignettes. Overall, therapy/counseling was perceived to be more helpful than medication for the OCD, AUD, and GAD vignettes, regardless of participants' reported description of the vignette characters' problems.

Our lack of significant findings for the relationship between participants' description of each vignette character's problems and perceived treatment helpfulness of medication and therapy/counseling are in part contrary to previous research (e.g., Iselin & Addis, 2003). One possible explanation for this contradiction is that the current study allowed participants to rate every treatment option that was presented to them. In other words, participants were not forced to rate the perceived helpfulness of either biological or psychological treatment options for each vignette, which allowed the authors to compare participants' ratings across various treatment options. These findings suggest that participants in the current study viewed therapy/counseling as helpful despite their beliefs about contributing factors for each disorder and despite being part of a culture that encourages pharmaceutical treatment options via direct-to-consumer advertising.

## Limitations and Future Directions

The current study has numerous strengths, such as the inclusion of multiple categories of psychiatric diagnoses depicted in our vignettes. While previous research on MHL has focused predominately on mood and anxiety disorders, we included a vignette depicting AUD, which historically has been understudied in relation to MHL. We recruited a large sample of undergraduate students and allowed them to rate every causal factor and treatment option that was presented in the study. Nevertheless, the current study is not without limitations. Our undergraduate student sample was quite homogeneous, with many participants identifying as White and as women. All participants were also recruited from Introduction to Psychology courses; content discussed in the course may have predisposed some participants to the problems described in the vignettes. Additionally, vignettes were presented in a fixed order, which may have influenced how participants viewed the seriousness of each disorder. For example, because the schizophrenia vignette was always presented before the GAD vignette, participants may have compared symptoms of psychosis to symptoms of generalized anxiety and rated the symptoms of generalized anxiety to be less severe. Future studies should consider presenting the vignettes in random order.

In conclusion, undergraduate students in the current study appeared to recognize a majority of the psychiatric diagnoses as a mental health problem, acknowledged the seriousness of the presenting problems, and recommended effective help-seeking behavior. Across the board, students endorsed therapy/counseling as the most helpful treatment compared to all other treatment methods. Students displayed the poorest MHL when attributing AUD to that of personal failings. This finding highlights the continued stigma associated with AUD and the need for further research on MHL as it relates to substance use disorders. Furthermore, many students failed to recognize AUD as a mental health issue; interestingly, however, this did not seem to discourage students from suggesting therapy/counseling as a helpful treatment option. While it is clear that undergraduate students could benefit from continued education on the contributing factors associated with AUD, it is encouraging to see a majority of students acknowledging the helpfulness of therapy/counseling for not only AUD, but for all of the psychological disorders studied herein.
